# Ursolic Acid Improves Intestinal Damage and Bacterial Dysbiosis in Liver Fibrosis Mice

**DOI:** 10.3389/fphar.2019.01321

**Published:** 2019-11-01

**Authors:** Si-Zhe Wan, Cong Liu, Chen-Kai Huang, Fang-Yun Luo, Xuan Zhu

**Affiliations:** Department of Gastroenterology, The First Affiliated Hospital of Nanchang University, Nanchang, China

**Keywords:** liver fibrosis, intestinal damage, bacterial dysbiosis, RhoA, ursolic acid

## Abstract

Liver fibrosis is a reversible process of extracellular matrix deposition or scar formation after liver injury. Intestinal damage and bacterial dysbiosis are important concomitant intestinal changes in liver fibrosis and may in turn accelerate the progression of liver fibrosis through the gut–liver axis. RhoA, an important factor in the regulation of the cytoskeleton, plays an important role in intestinal damage. We investigated the effects of ursolic acid (UA), a traditional Chinese medicine with anti-fibrotic effects, on intestinal damage and bacterial disorder through the RhoA pathway. UA treatment reduced intestinal damage by inhibiting the inflammatory factor TNF-α and increasing the expression of tight junction proteins and antibacterial peptides to protect the intestinal barrier. Moreover, the corrective effect of UA on bacterial dysbiosis was also confirmed by sequencing of the 16S rRNA gene. Potential beneficial bacteria, such as the phylum Firmicutes and the genera *Lactobacillus* and *Bifidobacterium*, were increased in the UA group compared to the CCl_4_ group. In liver fibrosis mice with RhoA inhibition *via* injection of adeno-associated virus, the liver fibrosis, intestinal damage, and flora disturbances were improved. Moreover, UA inhibited the expression of RhoA pathway components. In conclusion, UA improves intestinal damage and bacterial dysbiosis partly *via* the RhoA pathway. This may be a potential mechanism by which UA exerts its anti-fibrotic effects and provides effective theoretical support for the future use of UA in clinical practice.

## Introduction

Liver fibrosis is a reversible process of extracellular matrix (ECM) deposition or scar formation after liver injury caused by various factors, such as viruses, schistosomiasis, and alcohol (Lee et al., 2015; Seki and Brenner, 2015). Hepatic stellate cell (HSC) to myofibroblast (MFB) transformation is recognized as the central event in the development of liver fibrosis (Lee et al., 2015). The normal liver architecture and functions are disrupted in the liver in the state of liver fibrosis, and continuous development can eventually develop into cirrhosis and even liver cancer, which greatly threatens human health (Thompson et al., 2015; Affo et al., 2017). Liver transplantation is currently the only effective treatment once fibrosis develops into end-stage liver disease but is limited by the high cost and the shortage of donors and is thus unable to fully meet the needs of patients. Liver fibrosis is an early pathological feature of chronic liver disease due to its reversible characteristics; therefore, the development of anti-fibrotic treatments is of great significance. There is a special anatomical positional relationship between the liver and the gut; the portal vein-collected venous blood from the intestines provides 75% of the blood supply to the liver. The multiple physiological processes that are dependent on the reciprocal interaction between the liver and the intestines highlight the critical functional relationship between these organs. Therefore, some pathophysiological changes during liver fibrosis, such as impairment of the immune system, inflammation, and bile acid metabolism disorders, affect the intestinal tract through the gut–liver axis, causing intestinal damage and destruction of the intestinal barrier (Palma et al., 2007; Assimakopoulos et al., 2012; Kalaitzakis, 2014). The intestinal barrier acts as an important physical barrier to the intestine. Once this barrier breaks, it cannot prevent contaminants in the intestinal cavity from entering the blood and inducing liver and systemic lesions.

The human gastrointestinal tract hosts 10∼100 trillion bacteria, including approximately 500∼1,500 different bacterial species (Lozupone et al., 2012). The composition of the bacteria varies by individual, age, gender, and diet (Turnbaugh et al., 2009; Claesson et al., 2012). In recent years, many studies have confirmed that the intestinal microbiota participates in many activities in the body, such as metabolic homeostasis, immunomodulation, and inflammation (Maynard et al., 2012; Tremaroli and Backhed, 2012). As research has progressed, changes in the microbiota have been found in diabetes, arthritis, kidney disease, and atherosclerotic cardiovascular disease (Garidou et al., 2015; Jie et al., 2017; Ticinesi et al., 2018; Doonan et al., 2019).

The intestinal microbiota also undergoes dysbiosis during liver cirrhosis, which is often accompanied by a decrease in beneficial bacteria and an increase in harmful bacteria (Fouts et al., 2012; Shackel et al., 2014). Bacterial endotoxin, known as lipopolysaccharide (LPS), is found in the outer membrane of gram-negative bacteria; once LPS reaches the liver through a gap in the intestinal barrier, it is identified by the immune system through the recognition of pathogen-associated molecular patterns (PAMPs) (Kumar et al., 2011), which then induce the release of proinflammatory cytokines (Akira et al., 2006) and in turn accelerates the development of liver fibrosis.

Ursolic acid (UA) is a natural pentacyclic triterpenoid derived from various plants, including apples, basil, cranberries, and peppermint, and it has been reported to possess many biological activities, including anti-oxidative, anti-inflammatory, anti-ulcer, antibacterial, antiviral, anti-tumor, anti-obesity, hypoglycemic, antihypertensive, lipid-lowering, and liver protection properties (Ikeda et al., 2007; Wang et al., 2011; Wu et al., 2011; Kim et al., 2015; Cargnin and Gnoatto, 2017). A previous study showed that UA could reverse liver fibrosis by inhibiting the activation of HSCs *in vivo* and *in vitro* (He et al., 2015; Wang et al., 2011). However, the improvement of intestinal and microbiota dysbiosis by UA and the mechanisms involved are not clearly defined in liver fibrosis. RhoA, an important factor regulating the cytoskeleton by participating in actin stress fiber formation and myosin contraction (Asanuma et al., 2006; Kim et al., 2006), is involved in the integrity of the intestinal barrier (Tong et al., 2013) and has been shown to be associated with a variety of digestive diseases (Citalan-Madrid et al., 2017; Li et al., 2018). Therefore, in the present study, we aimed to determine the effects of UA on intestinal damage and microbiota dysbiosis in CCl_4_-induced liver fibrosis mice.

## Materials and Methods

### Experimental Animal Model and Design

**Figure 1:**
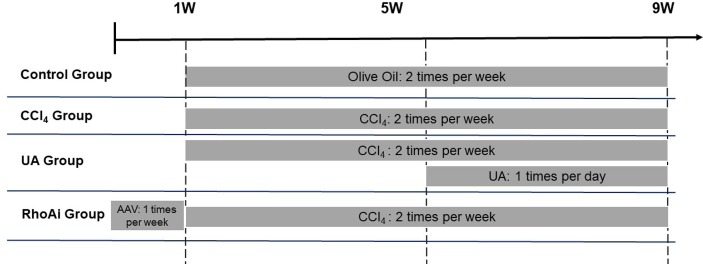
Flow diagram depicting the treatment of mice in all groups.

All wild-type (WT) C57BL/6 mice, obtained from the Department of Laboratory Animal Science of Nanchang University, were used for experiments. Mice were breed in an environment with a 12:12-h light/dark cycle, a room temperature of 22 ± 2°C, and 55 ± 5% humidity. WT mice weighing 20 to 30 g were randomly divided into five groups as follows (*n* = 8/group) (**Figure 1**): a control group was treated with olive oil (2 ml/kg) by gavage twice a week for 8 weeks (control group); liver fibrosis mice were induced by gavage of carbon tetrachloride (CCl_4_) (20% olive oil dilution, 2 ml/kg) twice a week for 8 weeks (CCl_4_ group); mice were randomly selected and treated. One group of mice that was gavaged with CCl_4_ for 4 weeks was then gavaged simultaneously with UA (40 mg/kg/day) for another 4 weeks (UA group). A group of mice received adeno-associated virus (AAV) *via* tail vein injection for 1 week to inhibit RhoA and then received CCl_4_ gavage twice a week for 8 weeks (RhoAi group) (**Supplementary Figure 1**). All procedures were performed according to the National Institutes of Health Guide for the Care and Use of Laboratory Animals. The experimental protocol was approved by the Animal Care and Use Committee of The First Affiliated Hospital of Nanchang University (Nanchang, China).

### Blood Index Test

An automatic biochemical analyzer was used to detect alanine aminotransferase (ALT), total bilirubin (TBIL), aspartate aminotransferase (AST), and triglyceride (TG) (Department of Clinical Laboratory, The First Affiliated Hospital of Nanchang University, China). The LPS content of serum was estimated spectrophotometrically using commercial diagnostic kits purchased from Elabscience (China).

### Histological Analysis

Liver and gut samples were fixed in 4% paraformaldehyde and cut into 5-µm sections for staining with hematoxylin and eosin (H&E), Masson’s trichrome staining, immunohistochemistry (IHC), TdT-mediated dUTP nick-end labeling (TUNEL), and immunofluorescence. H&E was used to observe the inflammatory cell infiltration of the liver and gut. We randomly selected five visual fields for observation, scored liver fibrosis using the METAVIR scoring system (Poynard et al., 1997) (**Table 1**), and evaluated intestinal mucosal damage using the Chiu scoring system (Chiu et al., 1970) (**Table 2**). IHC was used to reflect the expression site and expression intensity of related proteins. Liver fibrosis was estimated by Masson’s trichrome staining. Liver section dual-immunofluorescence was used for the simultaneous observation of apoptosis of hepatocytes and expression of α-SMA (Abcam, Cat. 5694, USA). Specimens incubated with the antibody were observed and photographed by confocal microscopy.

**Table 1:** METAVIR-based liver fibrosis scoring system.

Fibrosis grade	Comment
F0	No fibrosis.
F1	The fiber area of the portal area is enlarged, but no septa are formed.
F2	The fiber area of the portal area is enlarged, and a few septa are formed.
F3	Numerous septa are formed without cirrhosis.
F4	Cirrhosis.

F, fibrosis.

**Table 2:** Chiu-based intestinal mucosal injury scoring system.

Grade	Comment
0	Normal intestinal mucosa.
1	Widening of the subepithelial space of the villus accompanied by capillary congestion.
2	Extension of the subepithelial space with moderate lifting of the epithelial layer from the lamina propria.
3	Massive epithelial lifting down the sides of the villi, and a few tips may be denuded.
4	Denuded villi with exposed lamina propria and dilated capillaries. Increased cellularity of lamina propria may be noted.
5	Lamina propria rupture, bleeding, and ulceration.

### Construction and Injection of Vectors for RhoA Inhibition

To inhibit RhoA *in vivo*, AAV type 9 expressing a short hairpin RNA (shRNA) directed at RhoA (AAV-shRhoA) was injected into mice through the tail vein. The plasmid was designed with green fluorescent protein (GFP) to serve as a carrier for shRNA. The titer of the final AAV-shRhoA was 3.5 × 10^12^ viral particles/ml in phosphate-buffered saline (PBS).

### Gut Microbiota Analysis

Genomic DNA was extracted from the feces of mice using a stool DNA kit (Omega, China) according to the manufacturer’s instructions, and purity was verified by 1% agarose gel electrophoresis (BioFROXX, China). Primers (338F 5-ACTCCTACGGGAGGCAGCAG-3 and 806R 5-GGACTACHVGGGTWTCTAAT-3) were used to amplify the bacterial V3–V4 region of the 16S rRNA gene. Pyrosequencing of the polymerase chain reaction (PCR) products was performed on an Illumina MiSeq Instrument (Majorbio, China). Alpha diversity (Shannon and Chao1 index), beta diversity, and community composition were calculated. Gut microbiota functional prediction was performed to generate the operational taxonomic unit (out) abundance table using PICRUSt. The Cluster of Orthologous Groups (COG) of proteins and Kyoto Encyclopedia of Genes and Genomes (KEGG) database were compared through the corresponding greengene id of each OTU to obtain the COG family and KEGG Orthology information corresponding to the OTU.

### Western Blot Analysis

The liver and ileal tissues were homogenized in radioimmunoprecipitation assay (Solarbio, cat. R0020, China) buffer with phenylmethanesulfonyl fluoride (Cell Signaling Technology, cat. 8553, USA). The concentration of total protein was determined using a bicinchoninic acid (BCA) assay kit (Tiangen, Beijing, China). Equal quantities of protein extracts were resolved via 6–2% sodium dodecyl sulfate–polyacrylamide gel electrophoresis and electrophoretically transferred to polyvinylidene fluoride membranes. The membranes were blocked in 5% non-fat milk and subsequently incubated overnight at 4°C with primary antibodies. Then, a secondary horseradish peroxidase-conjugated anti-rabbit or anti-mouse IgG antibody (ZSGB-BIO, China) was applied, and images were developed via an enhanced chemiluminescence (ECL) detection kit (Thermo Fisher Scientific, USA).

### Quantitative Real-Time Polymerase Chain Reaction Analysis

Liver or ileal tissue was homogenized in 1 ml of TRIzol (Life Technologies, USA), and total RNA was extracted. The integrity of RNA was verified *via* agarose gel electrophoresis, and RNA was converted to cDNA using a FastQuant RT kit (Tiangen, cat. KR106-02, China). For qRT-PCR, SuperReal PreMix Plus (Tiangen, cat. SYBR Green, China) was used to determine the quantitative expression of RNA. The number of amplification cycles was 41. GAPDH was the reference gene. The qRT-PCR primers are shown in **Supplementary Table 1**. The mRNA levels of type I collagen, MMP1, and TIMP1 were normalized to GAPDH mRNA levels.

### Statistical Analysis

For the biochemical assays and histology score results, Image Pro Plus 6.0 software was used to check for normality. SPSS 23.0 software was used for data analysis. Image production and output were performed using GraphPad Prism 7.0 software. Each experiment was repeated three times to ensure confidence in the results. One-way analysis of variance (one-way ANOVA), Student’s *t* test, Mann-Whitney rank sum test, or Kruskal–Wallis *H* test was used to analyze the significant differences between groups. *P* < 0.05 was considered significant.

## Results

### UA Ameliorates Liver Injury and Fibrosis Induced by CCl_4_

**Figure 2:**
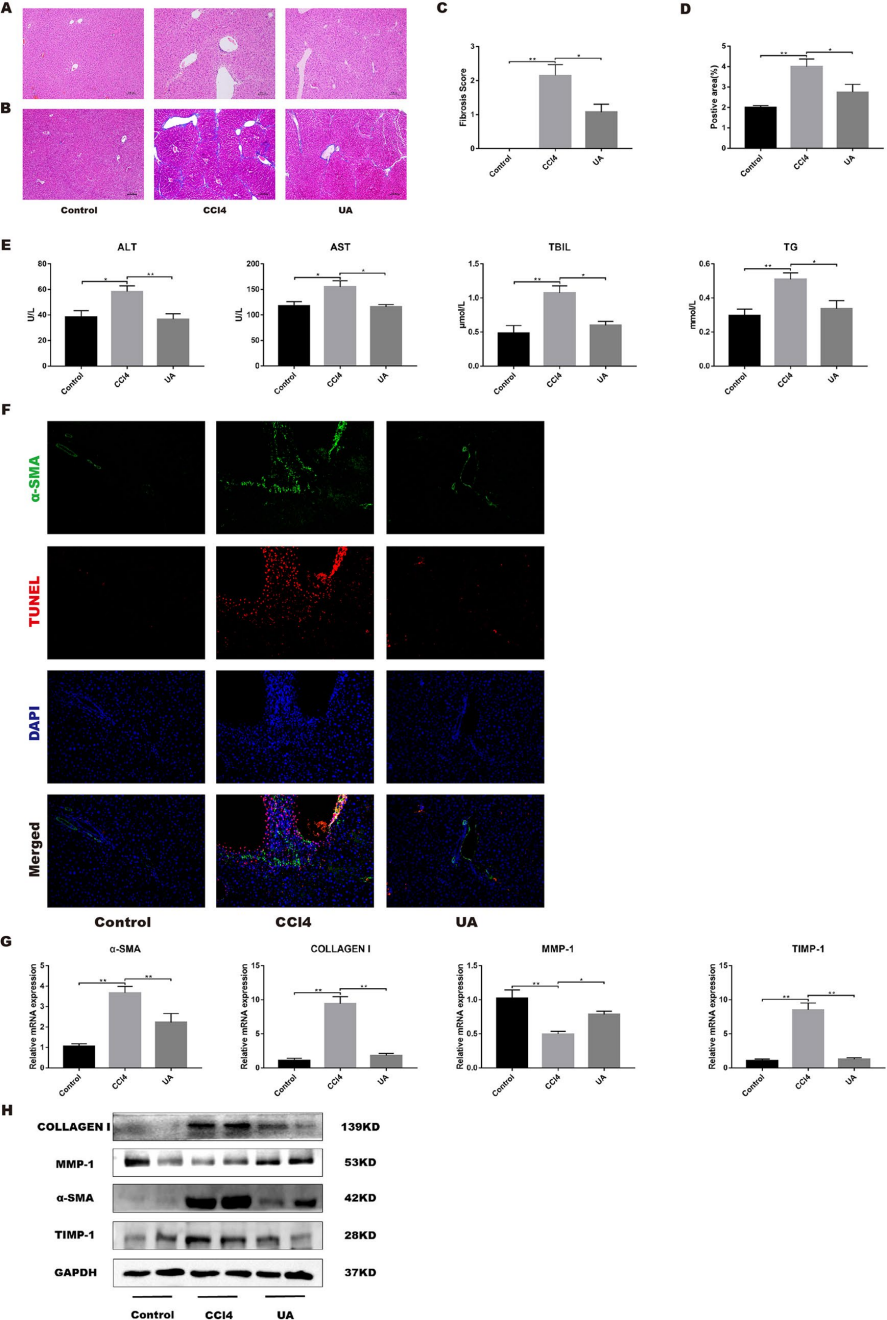
Effect of UA on CCl_4_-induced liver injury and fibrosis. **(A)** Hematoxylin and eosin (H&E) staining (100× magnification). **(B)** Masson’s trichrome staining (100× magnification). **(C)** Morphometrical analysis for fibrotic score. **(D)** Semi-quantitative determination of fibrosis by Masson’s trichrome staining. **(E)** ALT, AST, and TBIL levels in mouse serum. **(F)** Dual-immunofluorescence staining of liver sections from control group or CCl_4_ group or UA group stained for nuclei (DAPI, blue), aHSC (α-SMA, green), and apoptosis (TUNEL, red), and merged images. **(G)** Hepatic mRNA levels of α-SMA, type I collagen, MMP1, and TIMP1 were measured by RT-PCR. Data represent the mean ± SD of values per group. *P 0.05 and **P 0.01. UA, ursolic acid; ALT, alanine aminotransferase; AST, aspartate aminotransferase; TBIL, total bilirubin; TUNEL, TdT-mediated dUTP nick-end labeling; RT-PCR, real-time PCR. **(H)** α-SMA, type I collagen, MMP1, and TIMP1 expression of proteins were detected by western blot.

To clarify the effect of UA on the regression of CCl_4_-induced liver injury, we performed H&E and Masson’s trichrome staining on liver sections (**Figures 2A, B**). The liver architecture of the control group was normal with intact lobule structure and with sinusoids and cords located in order around central veins. However, CCl_4_ treatment caused destruction of the normal structure, the formation of a fibrous septum, and formation of a pseudo-lobe, accompanied by inflammatory cell infiltration and hepatocellular necrosis in the liver of mice. This finding indicates the formation of liver fibrosis and inflammatory infiltration in mice of the CCl_4_ group. Compared with that in the CCl_4_ group, histological liver injury, disorder in the hepatic lobular structure, fibrous space, and collagen deposition in liver tissue and inflammatory cell infiltration were alleviated in the UA group. As shown in **Figures 2C, D**, quantitative analysis showed a significant improvement in the fibrotic score and area (*P* < 0.05). We also performed serum liver function tests to assess the liver damage (**Figure 2E**). ALT, AST, TBIL, and TG levels in mouse serum results indicated deterioration of liver function caused by CCl_4_ treatment but were partly restored by UA treatment.

Activated HSC leads to reprogramming of liver metabolism, increased autophagy, and increased parenchymal cell damage, leading to loss of HSC retinoids and increased contractility, and amplification of growth factors and inflammatory signaling factors in the liver microenvironment, which in turn generates a large amount of ECM to promote the occurrence and development of fibrosis (Lee et al., 2015). Therefore, we investigated the effect of UA on activated HSCs *in vivo* by dual-immunofluorescence staining for α-SMA and TUNEL. As expected, α-SMA-positive cells, a biomarker of activated HSCs (Iredale et al., 1998), and apoptosis in hepatocyte were pronounced observed in the CCl_4_ group compared to the control group. In contrast, α-SMA-positive cells and apoptosis in hepatocyte were markedly decreased in the liver tissue of UA-treated mice (**Figure 2F**). Moreover, the change in the expression level of α-SMA also corresponded with the staining results (*P* < 0.05) (**Figures 2G, H**), thus clearly revealing that UA inhibits HSCs activation and reduces hepatocyte apoptosis in liver fibrosis mice.

The effect of UA on the expression of related factors involved in liver fibrosis in liver tissue was tested. At the mRNA level, CCl_4_ treatment induced high expression levels of type I collagen and TIMP1, but MMP1 promoted the degradation of the ECM and reduced fibrosis (Leroy et al., 2004), showing a decrease of expression. After UA intervention, the expression of type I collagen and TIMP1 genes was reduced (**Figure 2G**). At the protein level, results similar to the mRNA expression level results were observed. Type I collagen and TIMP1 showed increases in mice in the CCl_4_ group. The expression of these proteins was lower in the UA group than in the CCl_4_ group (**Figure 2H**).

### UA Improves Intestinal Injury and Integrity in Liver Fibrosis Mice

**Figure 3:**
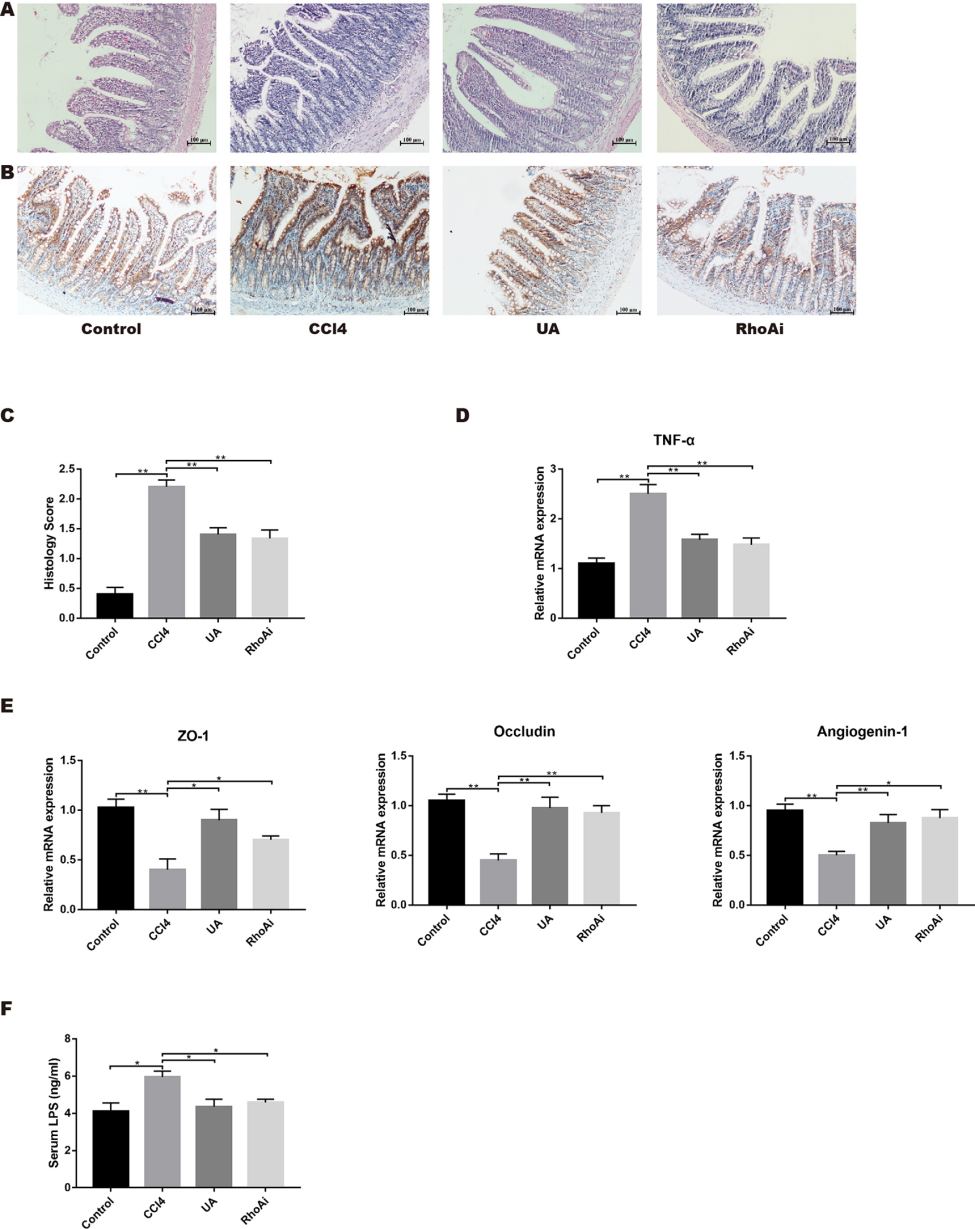
Intestinal damage in CCl_4_-induced liver fibrosis mice is attenuated by UA. **(A)** H&E staining (100× magnification). **(B)** TNF-α expression was measured *via* immunohistochemistry (IHC) staining (100× magnification). **(C)** H&E staining intensity was quantified by histomorphometry. **(D)** Ileal mRNA levels of TNF-α were measured by real-time RT-PCR. **(E)** Ileal mRNA levels of tight junction (TJ) proteins, ZO-1 and occludin, and intestinal antimicrobial peptides angiogenin-1 were measured by RT-PCR. (F) Serum LPS levels were determined by ELISA analysis. Data represent the mean ± SD of values per group. *P 0.05 and **P 0.01. UA, ursolic acid; RT-PCR, real-time PCR; LPS, lipopolysaccharide; ELISA, enzyme-linked immunosorbent assay.

Intestinal damage and destruction of intestinal barrier integrity are often accompanied by liver fibrosis; therefore, we studied the improvement of intestinal function by UA. Histological changes and inflammatory conditions in all groups were assessed by H&E staining (**Figure 3A**). The orderly uniformly distributed villi and intestinal glands were aligned in the ilea of the control group. After CCl_4_ treatment, the villi were disordered and fragmented, and gland lumens were larger than those in the control group. However, in contrast to the CCl_4_ group, the intestinal villi of the mice in the UA group were more orderly and regular (**Figure 3C**). We used IHC to detect the proinflammatory factor TNF-α in ileal tissue (**Figure 3B**). The expression of TNF-α in ileal intestinal epithelial cells was significantly increased in the CCl_4_ group. After UA treatment, the expression of TNF-α decreased in the intestinal epithelial cells. The expression of TNF-α was also tested in the intestinal tissue. The expression level of TNF-α increased in CCl_4_-treated mice. Compared to the CCl_4_ group, the level of TNF-α in the ileum of the mice in the UA group was decreased (*P* < 0.01) (**Figure 3D**). These results indicate that UA can significantly improve the damaged intestine and inhibit inflammation.

Destruction of the intestinal barrier is another important intestinal change in liver fibrosis. Intestinal barrier integrity is essential to resisting bacterial translocation and protecting the liver through the gut–liver axis (Cani and Delzenne, 2011; Schnabl and Brenner, 2014; Odenwald and Turner, 2017). Existing research has confirmed that tight junction (TJ) proteins and antibacterial peptide expression are inhibited by abnormal bile acid metabolism in fibrotic animals (Ubeda et al., 2016). Therefore, TJ proteins and antibacterial peptides in intestinal tissue were detected as important markers of the intestinal barrier. We speculated that UA protects the intestinal barrier by enhancing these connections. To verify this hypothesis, we explored the expression of the TJ proteins ZO-1 and occludin, and intestinal antimicrobial peptides, angiogenin-1. The expression of both TJ proteins and antimicrobial peptides was lower in the mice of the CCl_4_ group. In fibrotic mice treated with UA, the reduced level of ZO-1 and occludin mRNA expression was significantly restored. The protein expression levels in the ileum were similar to those of gene expression levels (**Figure 3E**). At the same time as the intestinal barrier is destroyed, the intestinal permeability is increased, and endotoxins enter the blood. Therefore, we measured the level of LPS in the serum. The level of LPS in the serum increased in the CCl_4_ group, which indicated that the intestinal permeability had increased in the CCl_4_-induced fibrotic mice. However, treatment with the UA combination reduced the LPS level in the intestines of liver fibrosis mice (*P* < 0.01) (**Figure 3F**), confirming the protective effect of UA and strengthening of the intestinal barrier to reduce intestinal permeation.

### UA Ameliorates Microbiome Dysbiosis in Mice With Liver Fibrosis

**Figure 4:**
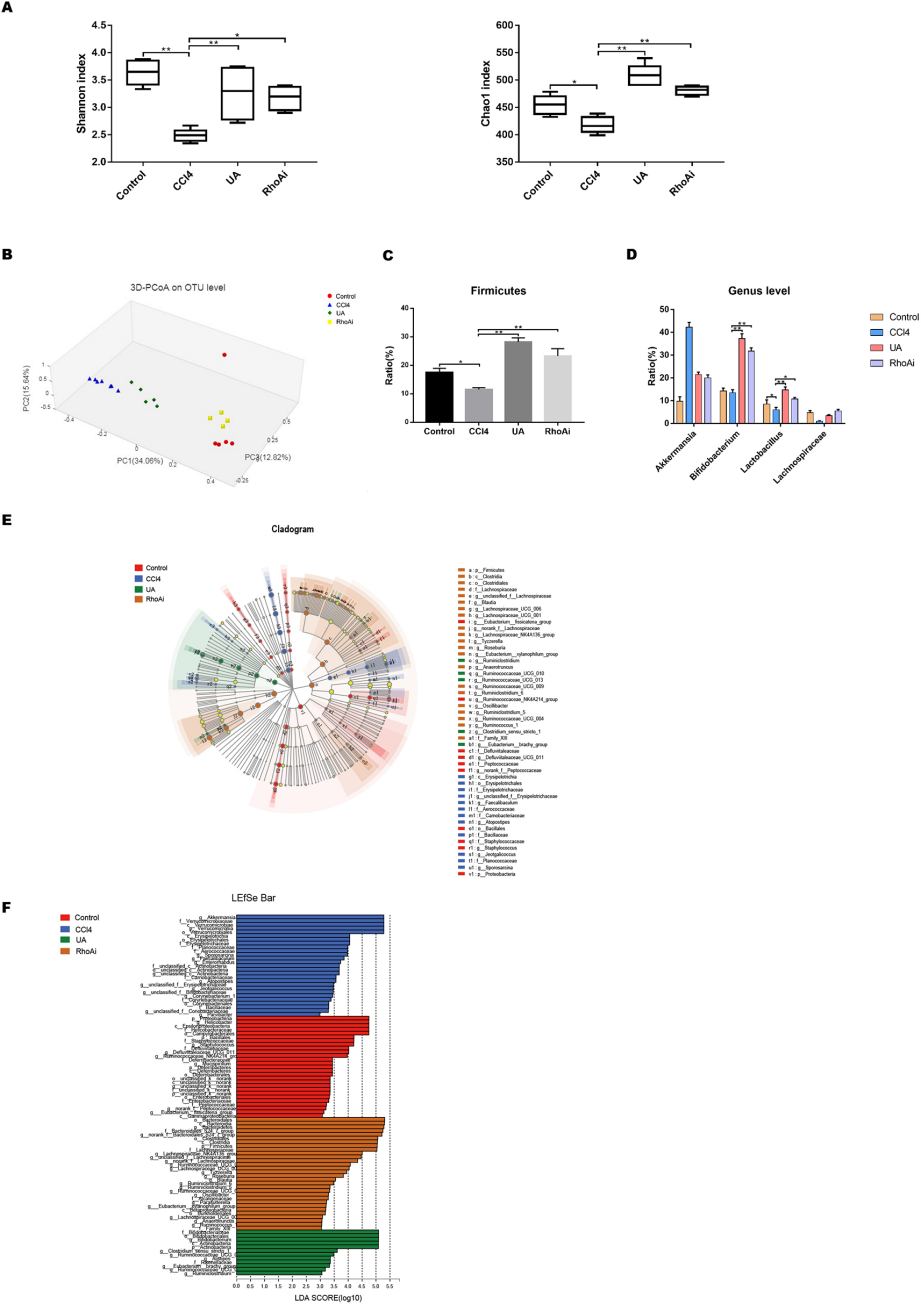
Effect of UA on intestinal microbiota dysbiosis in CCl_4_-induced liver fibrosis. **(A)** The alpha diversity of each group was obtained using the Shannon index and the Chao1 index. **(B)** Principal coordinates analysis (PCoA) for the weighted UniFrac distance of the intestinal microbiota. **(C)** At the phylum level, changes in Firmicutes in mice of each group. **(D)** At the genus level, changes in microbiota in mice in each group. **(E)** Linear discriminant analysis effect size (LEfse) prediction was used to identify the most differentially abundant bacteria in each group. **(F)** LDA scores showed significant bacterial differences in each group. Only the bacteria meeting a significant LDA threshold value of 2 are shown. Data represent the mean ± SD of values per group. *P 0.05 and **P 0.01. UA, ursolic acid; LDA, linear discriminant analysis.

Liver cirrhosis often affects intestinal microbiota. Here, we explored the changes in intestinal microbiota in a liver fibrosis mouse model and the improvement effect of UA. We investigated the change of microbiota by next-generation sequencing in all groups. First, we assessed some indices used to represent the variation in the diversity and abundance of the microbiota (**Figure 4A**). The value of the Shannon index, the estimated microbial diversity, was significantly lower in the CCl_4_ group than in the control group (*P* < 0.01). Compared with the CCl_4_ group, UA significantly restored this reduced value of the Shannon index and increased the diversity (*P* < 0.01). Similarly, the overall microbial abundance was significantly elevated in mice treated with UA compared with CCl_4_-induced mice based on the Chao1 index (*P* < 0.01). Next, the principal coordinates analysis (PCoA) plot showed a shift in the overall gut microbiota between the control group, CCl_4_ group, and UA group; each group belonged to a different microbiota (**Figure 4B**). These results demonstrate the changes in the microbiota during liver fibrosis and the corrective effects of UA.

The composition of the intestinal flora also changed due to the influence of liver fibrosis. We measured the composition of the microbial community in mice of all groups. At the phylum level, CCl_4_-treated mice displayed a reduced OTU level of Firmicutes, which include beneficial bacteria such as *Lactobacillus. Lactobacillus* can protect the body in multiple ways (Malik et al., 2018; Riehl et al., 2018; Caminero et al., 2019) (**Figure 4C**). However, UA ameliorated the distinctive enteric microbiome of mice with liver fibrosis. Compared with the CCl_4_ group, the UA-treated liver fibrosis mice showed higher OTUs of Firmicutes (*P* < 0.05), especially *Lactobacillus*. In addition, UA also had a similar improvement on bacterial community abundance at the genus level, including on *Bifidobacterium* and Lachnospiraceae (**Figure 4D**). These bacteria also serve to maintain health (Nicolucci et al., 2017; Schroeder et al., 2018). Next, the linear discriminant analysis effect size (LEfSe) was used to analyze the composition of the microbiota, and similar results were obtained. LEfSe showed that compared to the CCl_4_ group, the phylum Actinobacteria and the genera *Bifidobacterium* and *Ruminiclostridium* were enriched in the UA group (**Figures 4E, F**). We also predicted the function of the microbiome through the COG and KEGG databases (**Supplementary Figure 2**). The enriched pathways of the microbiome in CCl_4_-treated mice were as follows: “lipid transport and metabolism,” “signal transduction mechanisms,” “infectious diseases,” “xenobiotics biodegradation and metabolism,” and “metabolism of terpenoids and polyketides.” However, after liver fibrosis mice were treated with UA, the enriched pathways of the microbiome changed to the following pathways: “carbohydrate transport and metabolism,” “cell growth and death,” and “enzyme families.”

### UA Downregulates the RhoA Pathway, Improving Intestinal Damage and Gut Barrier Function

**Figure 5:**
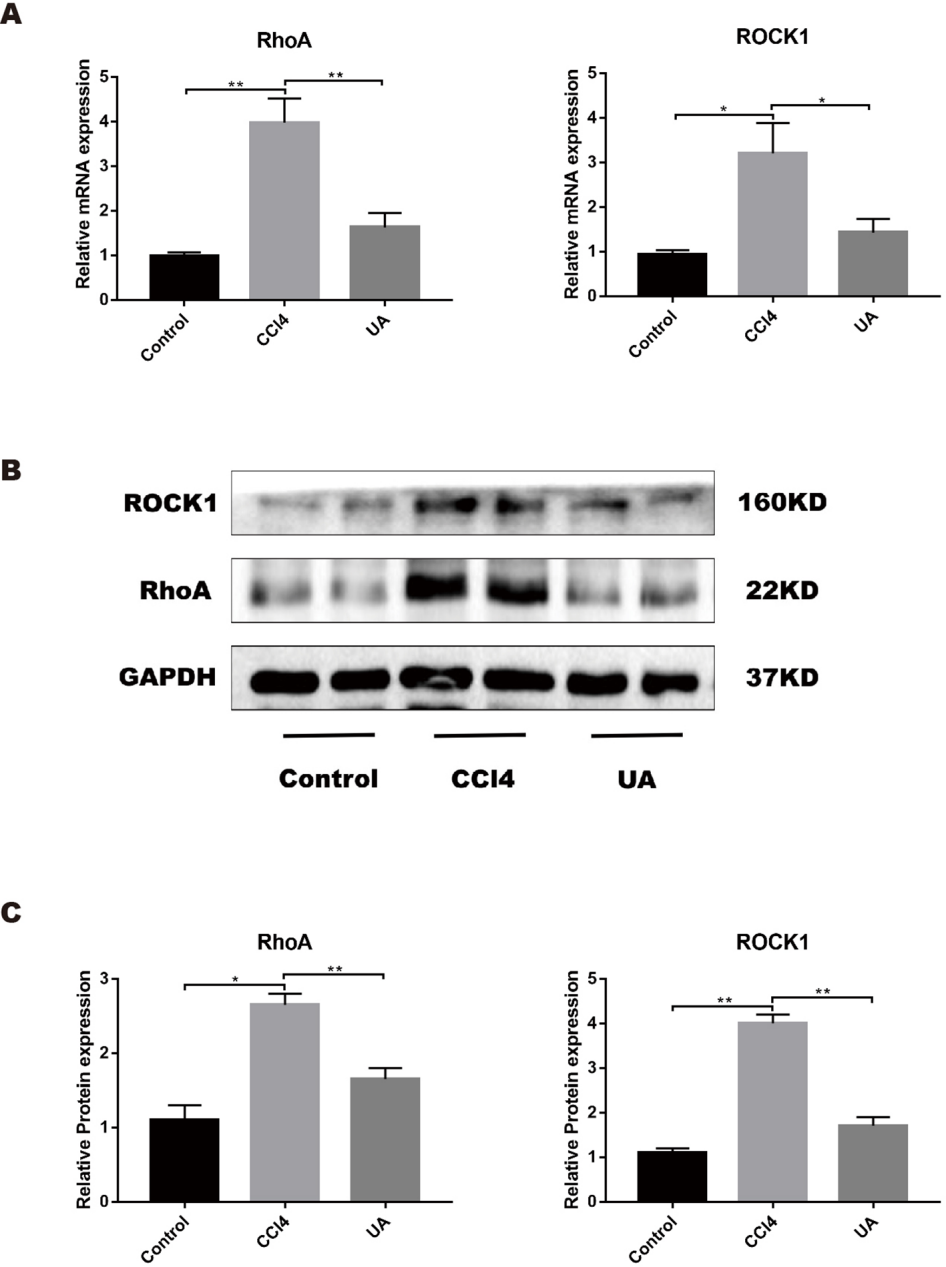
Effect of UA on the RhoA-related pathway in the ileum of liver fibrosis mice. **(A)** Ileal mRNA levels for RhoA and ROCK1 were measured by RT-PCR. **(B)** RhoA and ROCK1 expression of proteins was detected by western blot. **(C)** Histogram analysis of the levels of RhoA and ROCK1. Data represent the mean ± SD of values per group. *P 0.05 and **P 0.01. UA, ursolic acid; RT-PCR, real-time PCR.

As a molecule closely related to intestinal function and structure, RhoA plays an important role in intestinal damage, barrier destruction, and inflammation (Segain et al., 2003; Lopez-Posadas et al., 2016; Lopez-Posadas et al., 2017). We examined the ileal RhoA signaling pathway in liver fibrosis mice and the changes caused by UA. The mRNA expression levels of RhoA and ROCK1, the downstream target of RhoA, were upregulated in the ileal of mice in the CCl_4_ group (*P* < 0.01) (**Figure 5A**). Similarly, the protein expression levels of RhoA/ROCK pathway components were significantly higher in the ilea of mice in the CCl_4_ group than in the control group (**Figures 5B, C**). To better determine whether UA plays a role in intestinal protection through the regulation of RhoA, we inhibited RhoA in mice *via* tail vein AAV virus injection. In the RhoAi group, the histological damage, inflammatory cell infiltration, and TNF-α expression in the ilea were significantly decreased compared with the CCl_4_ group (**Figures 3A–D**). Furthermore, in RhoA-inhibited liver fibrosis mice, the destruction of the intestinal barrier integrity was also decreased (*P* < 0.05) (**Figure 3E**). Compared to the CCl_4_ group, the serum level of LPS was decreased in the RhoAi group, indicating the importance of RhoA in intestinal damage (**Figure 3F**).

We also studied the effect of UA on the expression of RhoA/ROCK1. UA treatment significantly decreased the induced ileal expression level of RhoA and ROCK in liver fibrosis mice (*P* < 0.01) (**Figures 5A–C**). From these results, we deduced that RhoA was probably the target of UA for intestinal protection.

### UA May Partially Improve Microbiome Dysbiosis Through RhoA

To explore whether UA corrects the microbiome dysbiosis and has a close relationship with RhoA, we compared the changes in the composition and proportion of intestinal microbiota of the UA group and RhoAi group. The Shannon and Chao1 indexes, reflecting the diversity and abundance of microbiota, in mice of the RhoAi group did not statistically significantly change compared to those of the UA group (**Figure 4A**). The PCoA plot showed that the composition of the microbiome was different between the groups. The plot indicated that the microbiota of mice in the RhoAi group was similar to that of the control group (**Figure 4B**). Moreover, an increased abundance of Firmicutes was found in RhoA-inhibited fibrotic mice. The abundance of beneficial bacteria, such as *Bifidobacterium* and *Lactobacillus*, in the RhoAi group was higher than that in the CCl_4_ group, although lower than the UA group (**Figures 4C, D**). Interestingly, LEfSe indicated that the RhoAi group was characterized by a preponderance of the phylum Bacteroidetes and the order Clostridiales, which is different from that of the UA group (**Figures 4E, F**). In addition, the results of microbiome function prediction showed that enriched pathways of the microbiome in the RhoAi group were as follows: “cell motility,” “transcription,” “carbohydrate metabolism,” and “immune system” (**Supplementary Figure 2**). We speculate that UA ameliorates the microbiota imbalance, which seems to partially occur through the regulation of RhoA.

## Discussion

In this study, we show that UA, a traditional Chinese medicine administered to liver fibrosis, markedly reduces intestinal injury and destruction of the intestinal barrier and improves intestinal dysbiosis. We explored the mechanisms involved in this effect, speculating that UA-induced intestinal and microbiota improvements may be achieved *via* RhoA-related signaling pathways.

Liver fibrosis is a pathophysiological process that often occurs in chronic liver disease. The continued development of liver fibrosis can eventually develop into end-stage liver disease. It has been reported that liver fibrosis is often prone to intestinal damage and destruction of the intestinal barrier in patients and animal models (Parlesak et al., 2000; Hartmann et al., 2012; Du Plessis et al., 2013). Our results confirmed that intestinal villi become sparse and damaged in fibrotic mice. The expression of inflammation-related factors such as TNF-α also increased, synergistically destroying the intestinal tract. TJ proteins, which are markers of intestinal barrier integrity, are important for the maintenance of intestinal integrity (Zeisel et al., 2018) and showed decreased expression in CCl_4_-treated mice. In this situation, intestinal pathogenic factors are more likely to enter the bloodstream and return to the portal vein, which may accelerate the development of liver fibrosis.

Recently, a series of studies have found that disorders of microbiota occur in many liver diseases, including alcoholic liver disease, non-alcoholic fatty liver disease, primary sclerosing cholangitis, and liver cancer (Mouzaki et al., 2013; Szabo, 2015; Yu and Schwabe, 2017; Tedesco et al., 2018). Patients with liver cirrhosis often have bacterial disturbances (Chen et al., 2011). Cirrhosis modifies bile acid composition and causes its metabolic disorders, as observed in patients, and in an animal model of cirrhosis induced by CCl_4_ (Lorenzo-Zuniga et al., 2003; Kakiyama et al., 2014). The intestinal microbiota is regulated by the bacteriostatic properties of bile acids, including the synthesis of antimicrobial peptides and modulation of innate immunity (Inagaki et al., 2006). However, there are relatively few studies on the changes in intestinal microbiota in response to liver fibrosis. Therefore, we sequenced the fecal microbiota of liver fibrosis mice through 16S rRNA technology. Our results are consistent with those of other studies in which the microbiota was found to be disordered in experimentally induced liver fibrosis animals (Wiest and Garcia-Tsao, 2005; Teltschik et al., 2012). By comparing the alpha diversity, Shannon and Chao1 indexes of the bacteria, we found a significant decrease in the diversity and abundance of mice in the CCl_4_-induced liver fibrosis group. The PCoA plot showed that the microbiota of the control group and the CCl_4_ group belonged to two different communities. Moreover, we also analyzed the composition of the mouse microbiome in each group. Firmicutes, a phylum of bacteria that has been proven beneficial to the body (Warner and Lolkema, 2003; Reijnders et al., 2016; La Rosa et al., 2019), appeared to decrease in the CCl_4_ group compared to the control group. Other beneficial bacteria showed similar changes at the genus level, such as *Lactobacillus* and *Bifidobacterium*. Interestingly, Proteobacteria, especially *Escherichia coli*, did not show significant changes between groups. The possible reason is that not all bacteria change dramatically in the early stage of liver fibrosis, and some bacteria may change with the increase of liver fibrosis. The dysfunctional microbiota can stimulate intrahepatic inflammation and LPS-induced toll-like receptor (TLR)-related pathway activation through the damaged intestinal barrier, aggravating liver fibrosis (Soares et al., 2010; Zhu et al., 2012).

Our previous research confirmed that UA can alleviate liver fibrosis in animal models (Gan et al., 2018). The mechanism may involve UA inhibiting HSC activation by mediating NOX4-related signaling pathways. Intestinal damage and bacterial dysbiosis are concomitant gut symptoms of liver fibrosis; however, the influence of UA on this is unknown. Therefore, we studied the effects of UA on the intestine and bacteria in liver fibrosis mice. In response to UA, intestinal damage was reduced, and intestinal barrier integrity was partially repaired in mice with liver fibrosis. Moreover, UA treatment restored damaged intestinal villi and inhibited the release of inflammatory factors. TJ proteins and antibacterial peptide expression showed some degree of recovery in the UA-treatment group. This recovery is significant for strengthening the intestinal barrier, which can prevent intestinal contaminants from flowing back into the liver through the portal vein.

A remarkable finding of our study was that UA can correct the intestinal microbiota imbalance in liver fibrosis mice. Previously, there has been little research on the effect of UA on the bacteria in liver fibrosis. We explored the improvement effect of UA on intestinal microbiota dysbiosis in CCl_4_-induced mice. After UA treatment, microbiota diversity and abundance partially recovered in liver fibrosis mice. Moreover, beneficial bacteria, including the phylum Firmicutes and the genera *Lactobacillus* and *Bifidobacterium*, rebounded in the UA group compared to the CCl_4_ group. These results confirm our hypothesis that UA restores microbiota stability.

RhoA, involved in cell migration, may be an important target for UA to improve intestinal damage and intestinal microbiota disorder. RhoA-related signaling pathways can induce TJ protein rearrangement and increase permeability by regulating myosin and actin, ultimately leading to the destruction of intestinal integrity (McKenzie and Ridley, 2007). Therefore, we used an AAV to interfere with the RhoA expression in a group of mice injected through the tail vein. Compared with the CCl_4_-treated mice, AAV-injected fibrotic mice had more less damage to the intestinal barrier and the intestinal microbiota, and the UA could inhibit the expression of RhoA pathway. These results indicate that the protective function of UA on the intestine is partly achieved by the inhibition of RhoA. It is worth noting that few studies have previously pointed out the link between RhoA and bacteria. In our study, we found that in RhoA-inhibited liver fibrosis mice, bacterial disorder was partly rescued. The mechanism involved may be that UA maintains the normal function of the intestinal mucosa by inhibiting RhoA and thus plays a role in correcting the disordered bacteria. This finding proves that RhoA may play an important role in the effect of UA.

The beneficial effect of UA on intestinal barrier integrity with the resultant reduction in disordered gut bacteria translocation may be a potential mechanism for UA to improve liver fibrosis. Liver fibrosis is often accompanied by the transfer of the microbiota to the portal vein and subsequent activation of the intrahepatic immune defense mechanisms, which causes an inflammatory reaction and accelerates the progression of liver fibrosis (Paik et al., 2003; Roh and Seki, 2013; Wiest et al., 2014). Therefore, we assume that UA prevents the bacteria from entering the vein by protecting the intestinal barrier, which reduces the immune response in the intestinal tract and the release of inflammatory factors, ultimately alleviating the development of liver fibrosis. This may be a potential mechanism by which UA exerts anti-fibrotic effects. However, the role of RhoA in intestinal microbiota dysbiosis and whether it is a target for UA intervention remain to be verified.

In conclusion, our research indicates that UA improves intestinal integrity and bacteria through RhoA-related signaling pathways, which provides strong evidence that UA indirectly contributes to the improvement of liver fibrosis through the gut–liver axis. The current study potentially provides new insights into the prevention and treatment of liver fibrosis by UA. However, the molecular mechanisms by which UA affects the gut and microbiota and the interaction with liver fibrosis need to be further explored. In combination with our previous research results, the findings herein provide theoretical support for the future use of UA in clinical practice.

## Data Availability Statement

All datasets generated for this study are included in the article/**Supplementary Material**.

## Ethics Statement

The animal study was reviewed and approved by Institutional Animal Care and Use Committee of the First Affiliated Hospital of Nanchang University (Nanchang, China).

## Author Contributions

S-ZW and CL contributed equally to this study. S-ZW and CL designed and wrote the manuscript. C-KH and F-YL analyzed data. XZ critically revised the manuscript.

## Funding

This study was supported by the National Natural Science Foundation of China (grant number 81660110), the “Gan-Po Talent 555” Project of Jiangxi Province, and the Nanchang University Graduate Innovation Special Fund Project (grant number CX2018205).

## Conflict of Interest

The authors declare that the research was conducted in the absence of any commercial or financial relationships that could be construed as a potential conflict of interest.
