# Supplementary material for: Ursolic Acid Improves Intestinal Damage and Bacterial Dysbiosis in Liver Fibrosis Mice
Source: Front Pharmacol. 2019 Nov 1;10:1321. doi: 10.3389/fphar.2019.01321 (PMC6838135; doi:10.3389/fphar.2019.01321)
Supplement: Supplementary file 1 [file Table_1.docx]

**Table 1 Primer sequences for RT-qPCR**

| PCR gene name | Forward primer (5′–3′) | Reverse primer (5′–3′) |
| --- | --- | --- |
| α-SMA | GTCCCAGACATCAGGGAGTAA | TCGGATACTTCAGCGTCAGGA |
| COLLAGENI | GCTCCTCTTAGGGGCCACT | CCACGTCTCACCATTGGGG |
| MMP1 | GCTGATACTGACACTGGTACTG | CAATCTTTTCTGGGAGCTC |
| TIMP1 | CCACAGATATCCGGTTCGGCTACA | GCACACCCCACAGCCAGCACTAT |
| TNF-α | CCAGGAGAAAGTCAGCCTCCT | TCATACCAGGGCTTGAGCTCA |
| ZO-1 | ATCGTTCCTAATAAGAACAGAGCC | GCTACGAAGACCTCGAAACC |
| Occludin | TCTTTGTATAAGTCACCGCCT | GAAGGTGTCTCTAGGTTATCGT |
| Angiogenin -1 | TAAGAATAAGCCAGTCTCCC | AGACTCATCAAAGTGGACAG |
| GAPDH | ATGGGTGTGAACCACGAGA | CAGGGATGATGTTCTGGGCA |
