# Supplementary material for: Ursolic Acid Improves Intestinal Damage and Bacterial Dysbiosis in Liver Fibrosis Mice
Source: Front Pharmacol. 2019 Nov 1;10:1321. doi: 10.3389/fphar.2019.01321 (PMC6838135; doi:10.3389/fphar.2019.01321)
Supplement: Supplementary file 2 [file Presentation_1.pdf]

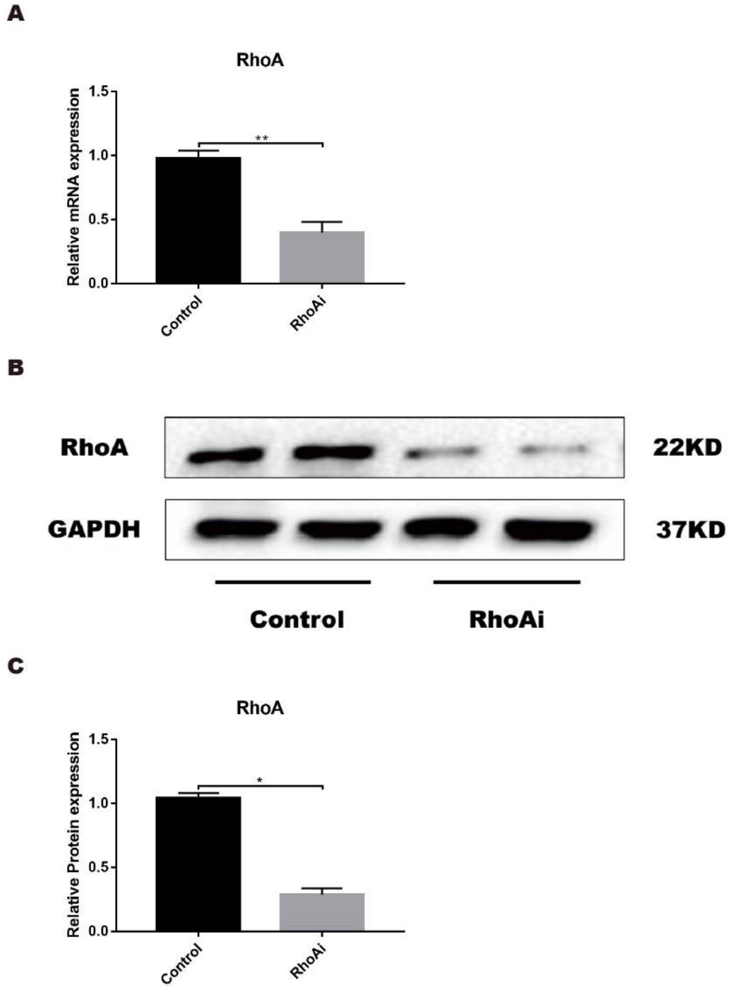

Supplementary Figure 1 Verification of the inhibition of the RhoA gene by adeno-associated virus (AAV) virus in mice. (A) Ileal mRNA levels of RhoA were measured by real-time RT-PCR. (B) RhoA expression of proteins was detected by western blot. (C) Histogram analysis of the levels of RhoA. Data represent the mean ± SD of values per group. *P < 0.05 and **P < 0.01.

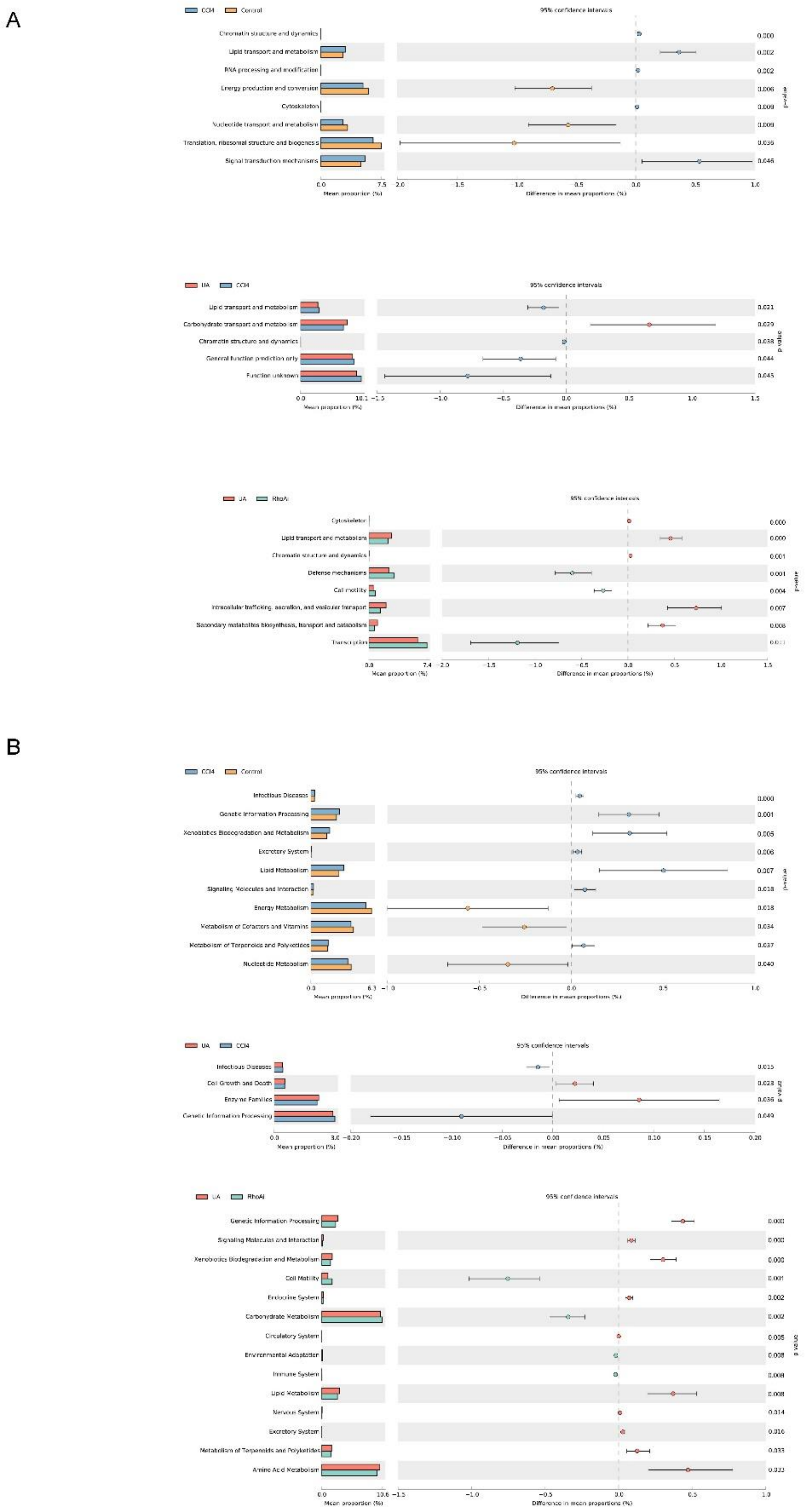

Supplementary Figure 2 Intestinal microbiota function prediction by comparing COG and KEGG databases. (A) COG analyses of significantly differential microbiome profiles among the control, CCl_4, UA and RhoAi groups. (B) KEGG analyses of significantly differential microbiomes among the control, CCl_4, UA and RhoAi groups.
